# Enhancement on the Tribological Properties of the Multilayer RGO/Al Matrix Composites by Cu-Coating Method

**DOI:** 10.3390/ma14123163

**Published:** 2021-06-09

**Authors:** Fengguo Liu, Ning Su, Renguo Guan

**Affiliations:** 1 School of Materials and Metallurgy, Northeastern University, Shenyang 110004, China; liufg@sylu.edu.cn (F.L.); ningsu@sjtu.edu.cn (N.S.); 2 School of Materials Science and Engineering, Shenyang Ligong University, Shenyang 110004, China

**Keywords:** multilayer reduced graphene oxide, tribological properties, aluminum matrix composites

## Abstract

Multilayer reduced graphene oxide (mrGO) was chemically modified by electroless plating of copper on surface to form mrGO-Cu. The scanning electron microscope (SEM) and transmission electron microscope (TEM) analysis revealed that nano-Cu particles were uniformly dispersed on the surface of mrGO. The mrGO-Cu powders were further utilized as reinforcements for aluminum (Al) matrix and the mrGO-Cu/Al composite was successfully fabricated through clad rolling of milled powder. The tribological properties of the mrGO-Cu/Al composites were explored. The tribological results show that the mrGO-Cu could reduce the friction coefficient and wear loss of mrGO-Cu/Al composites, since the mrGO-Cu participated in lubricating processes due to the formation of a transfer layer on the contact surface. Furthermore, it is found that the composition of mrGO-Cu could significantly influence the tribological properties of the mrGO-Cu/Al composites. The composites with 4% of mrGO-Cu for composites exhibited the best tribological behavior, which transformed from adhesive wear to abrasive wear, due to the formation of a graphite lubricating film.

## 1. Introduction

Aluminum and aluminum alloys have been widely used as light structural materials in aerospace, automotive and machinery manufacturing engineering as well as in other fields, due to their outstanding properties, such as high specific strength and good ductility [1]. However, the adhesiveness of aluminum composites may cause poor wear properties, which is a significant problem for the fabrication and application of the composites. Many efforts have been made to improve the abrasive resistance of Al-based composites [2,3,4].

As carbon materials can lead to a conformal protective coating on the sliding contact interfaces owing to low shear stress of C-C layer and self-lubricating property, reinforcing aluminum composites with carbon materials is regarded as an effective way to improve its abrasive resistance performance. Various types of carbon materials, such as carbon nanotubes, carbon fiber and graphite, have been applied as the reinforcements in aluminum alloy to improve their wear properties [5,6,7].

Since graphene was fabricated and observed by UK Geim and Novoselov by micro-mechanical exfoliation method on graphite, its excellent physical and electrical properties have been widely explored in science and industry [8,9,10,11,12]. Recently, many literatures showed that graphene could be used as reinforcements to improve the wear resistance of composites [13,14,15,16]. For example, Han Wang et al. [17] found that the tribological performance of poly vinyl chloride (PVC) composites could be improved obviously through adding multi-layer graphene (MLG), due to the enhanced toughness of the MLG/PVC composites and high self-lubricant properties of the MLG. Manuel Belmonte et al. [18] revealed that graphene nanoplatelets can enhance the tribological performance of ceramics due to the exfoliation of nanoplatelets that creates an adhered protective tribo-film. Jian Xuezhen et al. [19] revealed that the addition of graphene changed the wear mode of graphene/PTEE composites from adhesive wear to a mechanism of adhesive wear and abrasive wear. Zhang Yubing et al. [20] found that wear resistance of Si_3_N_4_ could be improved through adding graphene. Most of the recent studies on graphene reinforcing tribological properties focused on organic polymer and ceramics [21,22,23,24,25,26], but there are few reports on tribological properties of metal-based composites reinforced by graphene such as aluminum composites. 

In this work, we tried to explore the effect of graphene on enhancing the tribological properties of aluminum. The multilayer reduced graphene oxide was chemically modified by electroless Cu plating, aiming to improve the wettability between aluminum and graphene. Expectedly, the mrGO-Cu/Al composite was successfully fabricated through clad rolling of milled powder. Moreover, the influence of mrGO-Cu on the tribological properties of mrGO-Cu/aluminum composites, as well as its wear behavior and wear mechanism, were investigated in detail.

## 2. Materials and Methods

### 2.1. Materials and Preparation

**Figure 1:**
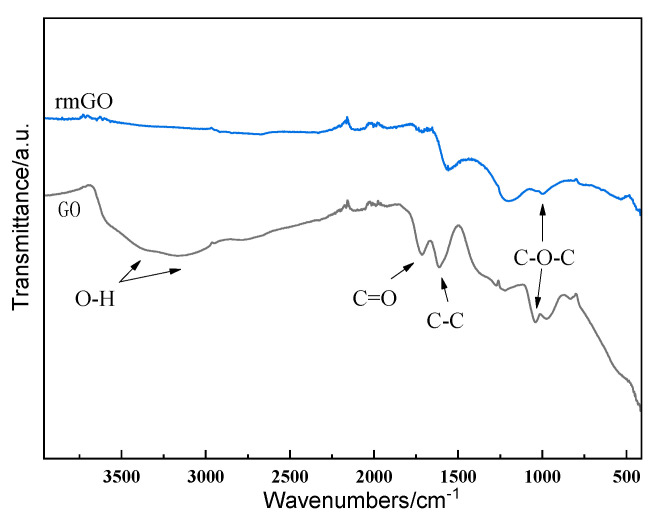
FTIR of GO and mrGO.

Pure aluminum power (≥99.5%) with an average particle diameter of 20 μm was used as the matrix. Multilayer reduced graphene oxide was prepared by Hummers method, and then modified with copper using an electroless Cu plating method [27]. Figure 1 shows the Fourier transform infrared spectroscopy (FTIR) results of the GO and mrGO. There were residual functional groups C-O-C in mrGO. Aluminum and mrGO-Cu powders were mixed for 1 h in alcohol solvent. Then, the mixed powder was vacuum dried for 12 h. The dried mixed powder was ball milled for another 30 min, and the ball-to-battery ratio was 7:1. Then, the homogeneous mixture was placed into aluminum tubes (48 mm in diameter), headed to 500 °C and incubated for 3 h. After that, the mixed powders were rolled with a deformation of 60–70% to obtain the mrGo-Cu/Al composite sheets. Finally, the composites were exposed by removing the surface aluminum layers. The pure aluminum specimen was prepared in the same way.

### 2.2. Characterizations

**Figure 2:**
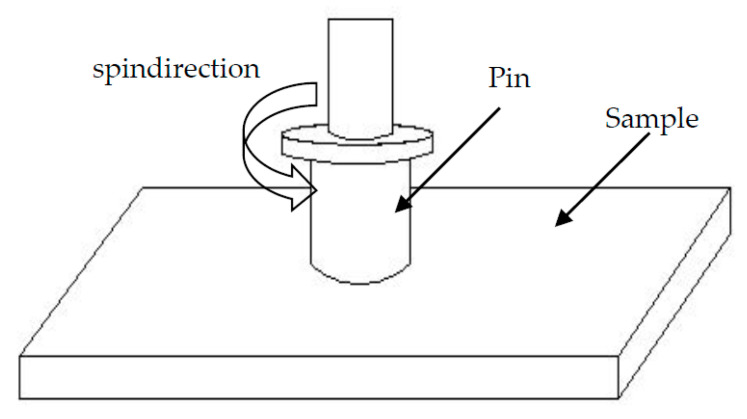
Schematic diagram of wear tests.

Pure aluminum powder and the mixed powder were analyzed by scanning electron microscopy (SEM; SSX-550, Shimadzu Corporation, Kotyo, Japan) coupled by energy dispersive X-ray spectroscopy (EDS). TEM (Transmission electron microscopy) was using to observe the graphene morphology. The tribological properties of the composites were measured using a MMW-1A configuration control universal wear testing machine (Jinan Yihua Friction Testing Technology Co., Ltd., Jinan, China) with a pin-on-disc apparatus, and friction pair and the disc are #45 steel and the mrGO-Cu/Al composites disk, respectively. The samples with size of 65 mm × 28 mm × 2.5 mm were polished with # 2000 sandpaper and treated by ultrasonic vibration in alcohol before wear test. The disc with hardness of HRC55 ~ 62, diameter of 30 mm and Ra = 3.2 μm was spun perpendicular to the sample surface, and the schematic diagram is shown in Figure 2. The friction and wear test was performed in an unlubricated condition under a load of 20 N with a rotation speed of 40 rpm. Friction coefficient is a function of friction time, which can be observed directly from the computer. The wear and friction coefficient experiment was carried out 3 times for each graphene content, and the results were averaged. After the test, the morphology of the worn surface was analyzed by scanning electron microscopy and EDS.

## 3. Result and Discussion

### 3.1. Microstructure of Composites

The morphology of the composite powder was observed by SEM and TEM to explore the distribution of mrGO-Cu and the state of copper particles on mrGO surface.

**Figure 3:**
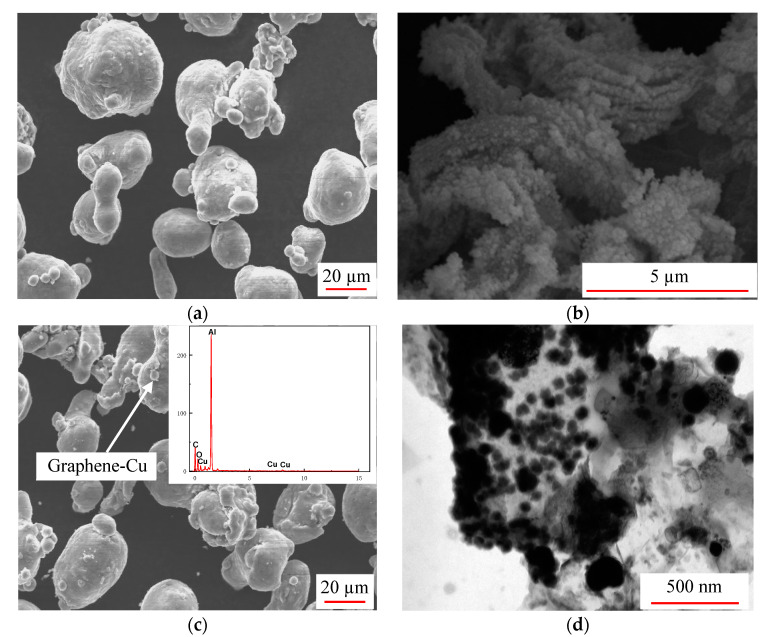
SEM images of (**a**) aluminum powder, (**b**) mrGO-Cu, (**c**) mixed composite powder and (**d**) mrGO-Cu after mixing.

Figure 3a shows SEM images of the pure aluminum powder with 2% content of mrGO-Cu. It can be seen that the pure aluminum powder has smooth surface with good dispersion. Figure 3b is the surface morphology of mrGO-Cu fabricated by chemical modification. It shows the nano-Cu particles covered on the surface of mrGO. However, the mrGO-Cu aggregated with each other because the connection of Cu particles or mrGO itself. Figure 3c is the morphology of mixed composite powder of pure Al with 2% content of mrGO-Cu, showing that the aluminum powder was evenly dispersed, but a small amount of aluminum powder agglomerated. Some substances were found on the surface of the aluminum powder, which contains C, Cu and Al elements (inset in Figure 3c). It can be concluded that the substance was mrGO-Cu. The TEM image in Figure 3d revealed that the aggregation was relieved and Cu particles were still coating the RGO surface after the mixing process. Due to the appearance of Cu coating on mrGO, the presence of Cu represents the location of mrGO.

**Figure 4:**
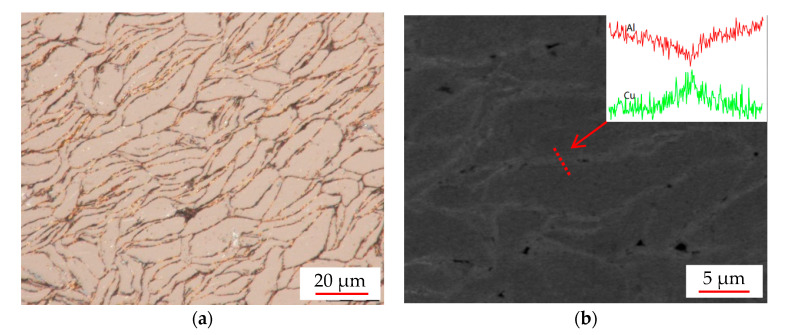
(**a**) Optical photomicrograph of mrGO-Cu/Al matrix composite, (**b**) SEM image of mrGO-Cu/Al matrix composite (insert is element distribution).

Figure 4 was the microstructure morphology of mrGO-Cu/Al composite. From Figure 4a, there was no Al_2_Cu precipitate in Al particles, which means that Cu is either distributed at the boundary or the solute in Al matrix. No aggregation of graphene was observed in Figure 4b, showing that the chemical modified mrGO-Cu (in Figure 3b) was exfoliated by the sheer force during the mixing process. The element distribution of Al and Cu, inserted in Figure 4b, shows that the content of Cu is increased while the content of Al is decreased. It is revealed that Cu was distributed uniformly around the boundary of Al particles. It can thus be regarded as the mrGO positions, considering that the mrGO was coated by Cu particles. 

### 3.2. Tribological Properties of Multilayer mrGO-Cu/Al Matrix Composites

**Figure 5:**
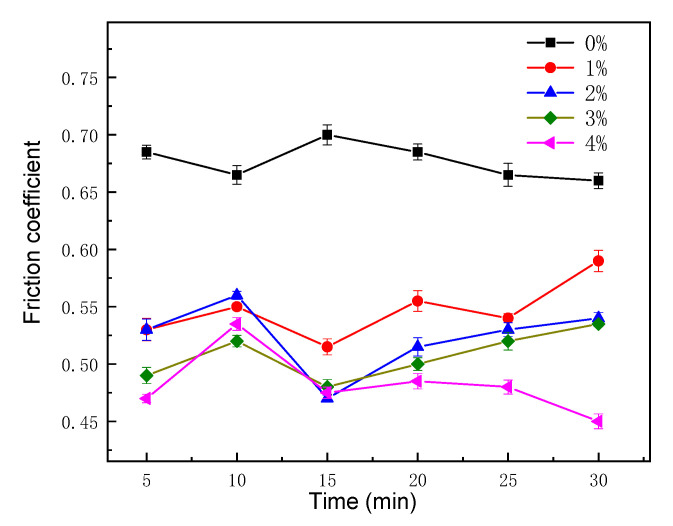
Friction factor of the composite with different mrGO-Cu contents.

The friction factor of the composite with different mrGO-Cu contents is shown in Figure 5. Compared with the pure aluminum substrate, the friction coefficient of aluminum composites is decreased with the addition of mrGO-Cu content. It could be seen that the friction coefficient of mrGO-Cu/Al composites obviously decreased when the treatment time for wearing was 25 min. Moreover, 1% content of mrGO-Cu can cause significantly dropping of the friction coefficient from 0.66 to 0.55 for the composites. When the content of mrGO-Cu was up to 4%, the friction coefficient of mrGO-Cu/Al composites reached 0.47 with a reduction of 28.8% compared to friction coefficient of pure aluminum matrix. The regular pattern was caused by the graphene lubrication film formed on the interface of friction pair. On one hand, the graphene lubrication film could change the mode from unlubrication friction to boundary friction. On the other hand, it could easily access the cavity of composites and decrease the roughness of friction surface, since graphene has a thin thickness. Therefore, there was a tendency towards decrease in the friction coefficient.

### 3.3. Effect of the Content of mrGO-Cu on Wear Loss

**Figure 6:**
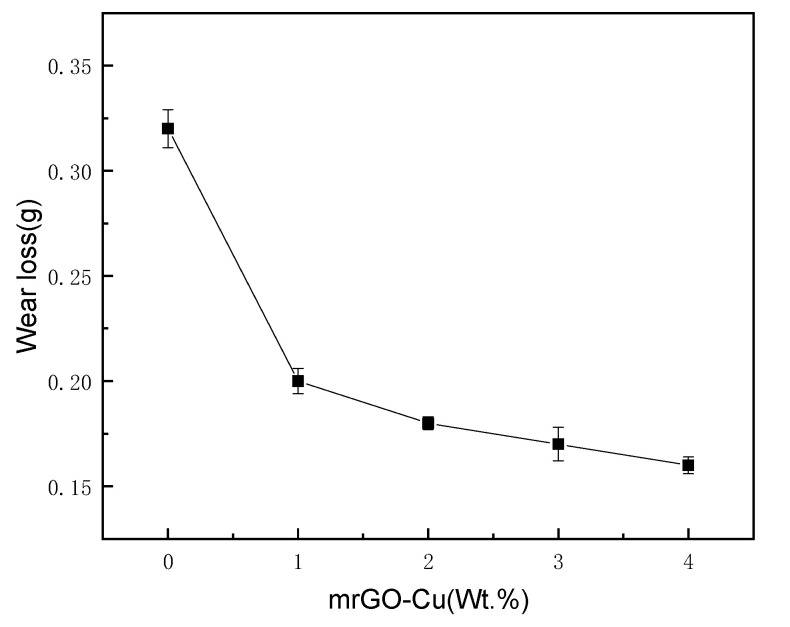
Wear loss of the composites with different contents of mrGO-Cu.

Wear loss of the composites with different contents of mrGO-Cu is presented in Figure 6. It could be found that the wear loss of mrGO-Cu/Al composites obviously decreased with the addition of mrGO-Cu. When the content of mrGO-Cu was up to 4%, the value of wear loss of composites (0.17) is the lowest, which is only 50% compared to that of pure aluminum matrix (0.34). The mrGO-Cu played a dispersion strengthening effect in the matrix alloy of the composite material. As the content of mrGO-Cu increased, the grain size of the composite material was refined and the hardness increased, the wear resistance of the material was improved. The wear loss of mrGO-Cu/Al composites decreased with the addition of mRGO-Cu may be interpreted as mrGO-Cu could form a lubricating film, and could hinder plastic flow of mrGO–Cu/Al composites, hence reducing plastic deformation and forming smaller scales flakes.

### 3.4. Effect of the Content of mrGO-Cu on Abrasion Mechanism

**Figure 7:**
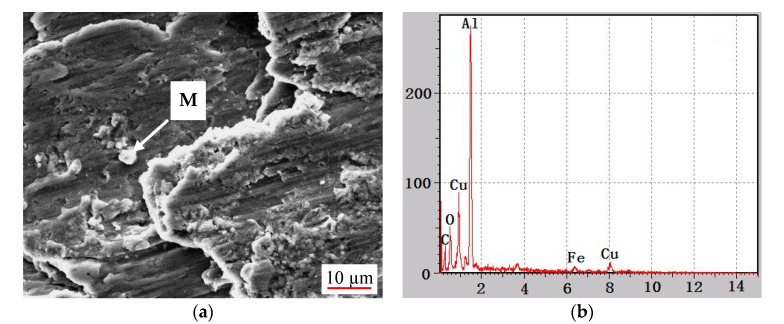
(**a**) SEM image of the composite worn surface, (**b**) Energy spectrum analysis of point M in (**a**).

SEM results with EDS analysis (Figure 7) were conducted on the worn surface of the mrGO-Cu/Al composites with 2 wt.% mrGO-Cu. It can be seen that the worn surface consists of Al, oxygen and copper elements. Research has shown that Al_2_O_3_ could easily be produced during the wear process. Therefore, it can be speculated that the white particles, as shown in Figure 7a, are mRGO-Cu and Al_2_O_3_. The presence of Fe (Figure 7b) was caused by grinding of #45.

**Figure 8:**
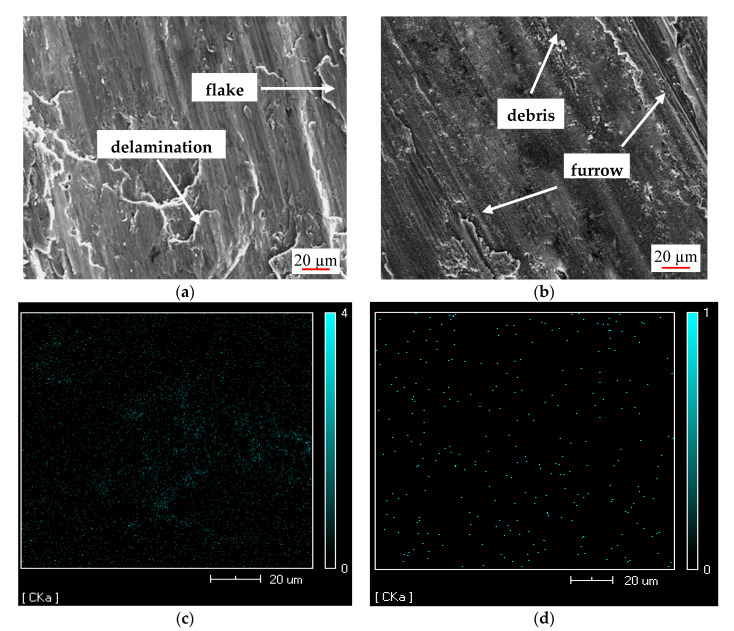
EDX images of worn surface with different mrGO-Cu contents (**a**) Al matrix, (**b**) 3 wt.% mrGO-Cu/Al, (**c**) Surface scanning image of worn surface of Al matrix, (**d**) Surface scanning image of worn surface of 3 wt.% mrGO-Cu/Al.

The worn surface morphologies of the sample with mrGO-Cu contents of 0 and 3 wt.% under the same conditions of friction are presented in Figure 8. On the surface of pure aluminum substrate it was possible to observe slipping, exfoliation, delamination and slight furrow as seen in Figure 8a. The friction behavior of high-speed motion produced a large amount of friction heat to soften the matrix alloy and caused adhesive wear, and the composite material was seriously worn [28]. Exfoliation and delamination are the typical adhesive wear morphologies. Oxide film on the matrix surface would be fractured under the effect of shear flow stress. Fracture process exposes the fresh metal in this case, the adhesive wear occurs with the direct contact of the two pure metal surfaces. Both broken oxide film and adhesive wear debris existed on the friction surface leading to slightly wear furrows. Therefore, adhesive wear and abrasive wear presented on the wear process [29]. In addition, the harder roughness of steel surface could also take some roles in the wear furrows owing to the indentation into the soft aluminum surface.

Compared to the morphology of pure aluminum substrate in Figure 8a, the wear morphology of sample with 3 wt.% mrGO-Cu shown in Figure 7b is smoother. Carbon element distribution analysis of samples in Figure 8a,b is shown in Figure 8c,d, respectively. From Figure 8d, it can be seen that as the sample with mrGO-Cu content of 3%, the graphene is fulfilled on the wear surface and tends to form a graphene film during the wear process. It may be interpreted by two reasons. Firstly, the multi-graphene layer was delaminated and transferred to the surface as multi-graphene has lower shear force. Secondly, graphene-Cu could be extruded by the plastic deformation force of Al substrate, thus the wear surface was covered by grapheme [30]. Because graphene film has an excellent self-lubricating property, the existence of graphite lubricating film made adhesive wear behavior weaken. Furthermore, mrGO-Cu nanosheets can inhibit the propagation of material cracks and reduce wear caused by pressure [31,32]. The large peeling sheets were almost invisible on the wear surface.

From Figure 8b, wide furrows and some debris can be found on the wear surface, indicating that although mrGO-Cu can be used as a lubricating film. However, the multi-graphite is modified by copper, both Cu and CuO, as micro-abrasive grains, could cut the soft Al substrate, and contributed to aggravating abrasive wear. At this stage, the abrasion mechanism was mainly abrasive wear.

## 4. Conclusions

Multilayer reduced graphene oxide was chemically modified by electroless plating Cu, and mrGO-Cu/Al composite was successfully fabricated through clad rolling of milled powder. The enhanced tribological properties of composites have been summarized as follows:

The graphene lubrication film could change the mode from unlubrication friction to boundary friction. With the increase in graphene content, the friction coefficient of composite materials gradually decreased. The decrement was obvious at a lower content of mrGO-Cu. Friction coefficient of the composite with 4% of mrGo-Cu could reduce by 28.8%, and the wear loss of the composite reduced by 50% compared to pure aluminum.

The mrGO-Cu played an important role of dispersion strengthening in the aluminum matrix. As the content of mrGO-Cu increased, the grain size of the composite material was refined and the hardness increased, the wear resistance of the material was improved. The wear loss of mrGO-Cu/Al composites decreased with the addition of mrGO-Cu.

Under the action of shear force, the mrGO-Cu was unfolded at the wear interface. The mrGO-Cu can form a graphite lubricating film and thus reduce the adhesive wear. Wear mechanism was also dominated by adhesive wear into abrasive wear with the addition of mrGO-Cu.

## Data Availability

Data sharing not applicable.
